# Involving patient in the early stages of health technology assessment (HTA): a study protocol

**DOI:** 10.1186/1472-6963-14-273

**Published:** 2014-06-20

**Authors:** Marie-Pierre Gagnon, Bernard Candas, Marie Desmartis, Johanne Gagnon, Daniel La Roche, Marc Rhainds, Martin Coulombe, Mylène Tantchou Dipankui, France Légaré

**Affiliations:** 1 Research Centre of the CHU de Québec, Hôpital Saint-François d’Assise, 10 rue de l’Espinay, D6-726, Quebec City, QC G1L 3L5, Canada; 2 Faculty of Nursing, Université Laval, Quebec City, QC, Canada; 3 National institute of public health, Quebec City, QC, Canada; 4 CHU de Québec, Quebec City, QC, Canada; 5 Department of Family Medicine, Université Laval, Quebec City, QC, Canada

**Keywords:** Public and patient participation, Research priorities, Health technology assessment, Public involvement, Knowledge translation

## Abstract

**Background:**

Public and patient involvement in the different stages of the health technology assessment (HTA) process is increasingly encouraged. The selection of topics for assessment, which includes identifying and prioritizing HTA questions, is a constant challenge for HTA agencies because the number of technologies requiring an assessment exceeds the resources available. Public and patient involvement in these early stages of HTA could make assessments more relevant and acceptable to them. Involving them in the development of the assessment plan is also crucial to optimize their influence and impact on HTA research. The project objectives are: 1) setting up interventions to promote patient participation in three stages of the HTA process: identification of HTA topics, prioritization, and development of the assessment plan of the topic prioritized; and 2) assessing the impact of patient participation on the relevance of the topics suggested, the prioritization process, and the assessment plan from the point of view of patients and other groups involved in HTA.

**Methods:**

Patients and their representatives living in the catchment area of the HTA Roundtable of Université Laval’s Integrated University Health Network (covering six health regions of the Province of Quebec, Canada) will be involved in the following HTA activities: 1) identification of potential HTA topics in the field of cancer; 2) revision of vignettes developed to inform the prioritization of topics; 3) participation in deliberation sessions for prioritizing HTA topics; and 4) development of the assessment plan of the topic prioritized. The research team will coordinate the implementation of these activities and will evaluate the process and outcomes of patient involvement through semi-structured interviews with representatives of the different stakeholder groups, structured observations, and document analysis, mainly involving the comparison of votes and topics suggested by various stakeholder groups.

**Discussion:**

This project is designed as an integrated approach to knowledge translation and will be conducted through a close collaboration between researchers and knowledge users at all stages of the project. In response to the needs expressed by HTA producers, the knowledge produced will be directly useful in guiding practices regarding patient involvement in the early phases of HTA.

## Background

Public and patient involvement is recognized as an effective approach to improving the relevance of research, increasing the value of scientific research without compromising its rigour, facilitating the implementation of innovations, and increasing the external validity of results [1,2]. In health technology assessment (HTA), public and patient involvement is also considered as a priority [3-8]. As direct beneficiaries of health services, patients have a comprehensive knowledge of the impact and the effects of a treatment or a technology on their condition and on different aspects of their life [5,9].

Several authors have noted that decisions for selecting technologies to assess should be more focused on the values and needs of patients and the public [4,5,10]. The selection of HTA topics which includes identifying technologies for assessment and prioritizing them, is part of the mandate of HTA units and agencies. Because the number of technologies requiring an assessment significantly exceeds the resources available, all HTA organizations face a problem of prioritization [11]. Some studies have shown that patients’ priorities differ from those of researchers and clinicians [2,12-15]. In the field of cancer, Corner and Wright [12] have demonstrated that although the biological aspects and those related to treatment were considered important by patients, they rated the management of practical, social and emotional issues as a higher priority. The results echoed those of an earlier study conducted by the U.S. National Cancer Institute in 1997 [13] showing that although patients and their families were in favour of cancer research, they felt that research often more clearly served the interests of clinicians and researchers than the priorities of the people directly affected [13]. Including patients’ experiential knowledge in the selection of HTA topics makes it possible to counterbalance the potential biases brought by scientists or health professionals [16,17]. By involving patients and the public in the identification and prioritization of HTA topics, assessments are more likely to be relevant to them and adapted to their needs [10,18].

A review of the different roles for patients and the public in HTA shows that many agencies worldwide give a role to patients and/or to the public in the identification of assessment topics [10]. Most of the time, a downloadable electronic form is available to members of the general public on the websites of HTA organizations, thus enabling them to suggest assessment topics [10]. A more proactive approach is also used by the program of the National Institute for Health Research (NIHR) in the United Kingdom [19-21]. This organization collaborates with community groups and the James Lind Alliance, a non-profit initiative funded by the NIHR, whose purpose is to ensure that publicly funded research corresponds to what matters to both patients and clinicians [22]. Community groups are invited to submit suggestions for research topics based on their experiences with particular health conditions. In the United States, the Patient-Centered Outcomes Research Institute also seeks questions from patients through a website, social media and engagement activities [23].

Regarding the prioritization of HTA topics, several agencies across the world, including the Canadian Agency for Drugs and Technologies in Health [24], are using similar criteria: the burden of disease associated with the technology, the potential clinical impact, the potential economic repercussions, the impact on the funding budget of the technology, the availability of evidence on the technology, and the presence of alternatives [11]. Other context-specific criteria are also considered, such as the expected level of public interest, the potential controversy of the topic, the accessibility and reimbursement of the technology, and the variation in the rates of use [24]. However, there is little knowledge available on how these criteria are actually used to guide decisions in the selection of HTA topics [11].

Although several researchers consider that the prioritization stage in HTA is value-laden and that a greater involvement of stakeholders (including patients) would be important [10,25], patients are mostly excluded from this stage in HTA [10], with some exceptions. One of these is the HTA program of NIHR in the United Kingdom (UK), which has established a whole infrastructure to support public involvement in the identification and prioritization of assessment topics [26,27]. Under this program, the public contributes to prioritization in two ways: as reviewers of vignettes that serve as the basis of discussion of the prioritization committees and as members of the committees themselves [10,21]. Another experience of public involvement in prioritization of HTA topics is that of the Centers for Medicare and Medicaid Services in the USA, which involve citizens in prioritization by offering them the opportunity to comment upon potential issues that are posted on their website [10].

**Figure 1:**
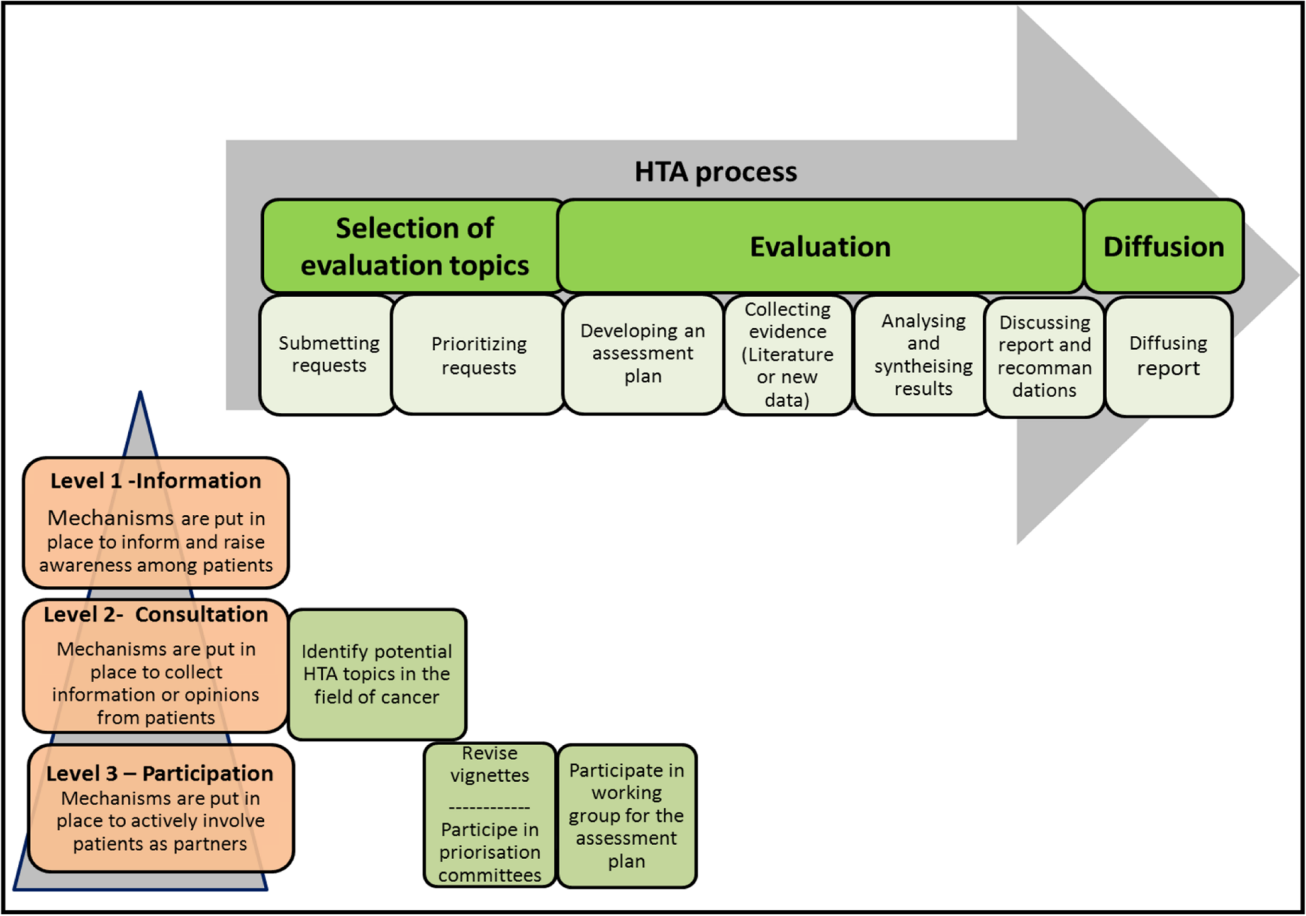
**Interventions to promote patient involvement in the early stages of the HTA process.** Legend: Levels of patient involvement according to the stages of the HTA process.

The development of the assessment plan related to the topic selected for HTA is also a stage at which it is particularly relevant to involve patients [28-30]. This stage, which includes the identification of relevant issues, dimensions, measures and indicators related to the topic selected, appears crucial to involve patients because it enables an optimizing of their influence and impact on the entire research initiative [4,5,29,30]. User involvement at this stage can favour the relevance of research questions to patients’ needs and ensure that the indicators and measurement instruments actually reflect the dimensions they want to include [14,20,28,30].According to our conceptual framework of patient involvement in HTA, three approaches concerning the mechanisms of patient involvement could be distinguished: active participation, consultation, and communication/information (see Figure 1). Each of these approaches is characterized by the level of patient involvement (participation as full partners, consultants, or recipients of information) and by a set of corresponding techniques or methods.

Mitton and collaborators [31] have reviewed the various techniques or methods of public involvement used in the prioritization and allocation of resources. Despite the lack of available evidence, their review suggests that using deliberative methods may be associated with more satisfactory results than using other methods that are more consultative and characterized by a lower level of public involvement [31]. Methods that involve face-to-face interaction between participants from the public and decision makers also generate greater participant satisfaction, both in terms of process and results [31]. Furthermore, a study by Abelson [32] demonstrates that the views of members of the public concerning priorities differ systematically when they are given the opportunity to discuss the topics. According to this study, deliberative methods could facilitate a more informed choice in the prioritization of topics than methods such as questionnaires or individual interviews, where the opinion of the public participants is requested only once and without first being discussed.

### Goal and setting of the project

This project builds upon a previous project entitled *Involving patients in HTA activities at local level: a study protocol based on the collaboration between researchers and knowledge users*[33] which allowed the involvement of patients in the HTA process of assessing alternatives to restraint and seclusion for hospitalized or institutionalized adults. Based on the results of this experiment, this new project will involve patients in HTA stages that have not been considered in our previous work, namely the identification and prioritization of HTA topics, and the development of the assessment plan of the prioritized topic.

HTA producers need to find effective ways to incorporate the perspective of patients in HTA structures and activities [3,25]. This project has been developed in collaboration with the HTA Roundtable of the Integrated University Health Network (IUHN) of Université Laval (Quebec, Canada) that was set up in order to support healthcare organizations throughout its territory in HTA and knowledge transfer. This HTA roundtable recently initiated a consultation among its member organizations (in a catchment area covering the whole of eastern Quebec) whose results showed that assessments could better meet local and regional needs. In Quebec, patients’ experience regarding their choice of treatments, technologies, and service modalities can differ depending on the region. Regions with a university hospital have easier access to specialized and subspecialized care, and new technologies are usually introduced earlier in these regions.

In continuity with our previous work, and to improve the consideration of regional and local needs, the HTA roundtable of this IUHN collaborated with researchers from the project in order to involve patients with other stakeholders from the six health regions of its catchment area in the identification and prioritization of HTA topics related to cancer. The theme of cancer was chosen by knowledge users of the team because it represents the leading cause of death in Canada [34], and the entire problem area raises a very broad array of questions and issues that encompass the full range of services and health policies, ranging from primary prevention, screening of target populations, access to healthcare services, and treatment and expensive drugs, to palliative and end of life care. Moreover, cancer represents a field in which new technologies, including drugs, evolve quickly, and for which indications of current technologies also expand rapidly, raising several relevant HTA questions.

The main study objectives are: 1) to set up interventions promoting patient involvement in these regions in the selection of HTA topics (identification and prioritization) and in the development of the assessment plan of the chosen subject, and 2) to assess the impacts of these interventions from the point of view of patients and other groups involved in HTA. In this project, the term patient refers to a person affected by a given technology (currently or in the past), but also includes patient representatives such as community groups.

## Methods

Patients will be involved in four specific activities of the HTA process (see Figure 1): 1) identification of HTA topics; 2) revision of vignettes that will be developed to inform the prioritization of the topics; 3) prioritization of HTA topics; and 4) development of the assessment plan. Different mechanisms of involvement will be used according to the level of involvement (consultation and direct participation) and the type of activity (topic suggestion in a web form, vignette review, deliberative meetings with other stakeholders to prioritize topics, HTA working group). The research team will observe and evaluate the process and outcomes associated with patient involvement in all these activities. A participatory evaluation approach will be used [35-38], involving stakeholders at all stages of the research process (planning, design, data collection and analysis, identification of outcomes, conclusions, recommendations, and dissemination of results).

### Identification of HTA topics

We will undertake consultations among various healthcare organizations and patient and community groups from the IUHN territory in order to identify potential topics for HTA in the field of cancer. With the collaboration of the CEOs of the regional health and social services agencies, the regional coordinator of oncology services, and community organizations in cancer collaborating to the project, we will contact healthcare professionals, managers and community organizations to invite them to suggest topics by way of an online or paper form. They will also be invited to diffuse information about the project in their community. We will provide instructions and specific examples on the suggestion form. Participants will have to indicate whether they are patients (current or past), close relatives of patients, professionals, or healthcare managers in order to allow for a consideration of the specificities of each of these groups.

### Filtration of topics and preparation of vignettes

Following the example of the NIHR HTA program in the United Kingdom [20], we will then undertake a filtration of the suggested topics. The topics will be examined by trained staff from the HTA unit of the Quebec University Hospital Centre (QUHC) whose members will check their relevance for an HTA program and whether they have already been covered by a previous HTA project. Moreover, we will set up a central prioritization committee assembling representatives of different categories of stakeholders (managers, healthcare professionals and patients) from the various regions covered by the project. This committee will have to validate recommendations made by the HTA unit. The six regions covered by the study will be divided into three groups: university, peripheral and intermediary, and remote [39]. As a patient representative will be recruited in each group of regions, at least three patient representatives will participate in the prioritization meeting in order to validate the final list of topics.

Based on the UK experience [20,40], we will then prepare short vignettes (less than one page) for each topic in order to provide preliminary information on the research question (technology, group of patients affected, and context) and the potential effectiveness of the technology. These vignettes will be sent to experts from relevant fields for comments. At least one patient representative, selected from those referred by patient organizations, will be asked to review the vignettes. Reviewers will comment upon the relevance and importance of the research and the knowledge that could be produced [19].

### Prioritization of HTA topics

We will organize deliberation sessions in each region category (university, peripheral or intermediary, and remote) with groups of approximately nine people – including healthcare professionals, decision makers and patients – to prioritize HTA topics. Based on the vignettes produced at the previous stage, participants will be invited to discuss the technologies presented. Two members of the project team, including an HTA producer, will facilitate the deliberation sessions. Finally, each participant will vote individually, and, in secret, will rate the proposed topics and classify them in priority order. The votes of the participants will be compiled according to the category to which they belong [41].

The central prioritization committee set up during the filtration stage will meet again to establish, through a deliberative method, a list of five topics in order of priority, as a means of identifying one priority topic that will be evaluated as well as other important topics that could be assessed in the future. The final decision as regards accepting the proposed HTA topic will revert back to the HTA Roundtable of Université Laval’s Integrated University Health Network, which could mandate the HTA unit of the QUHC and its partners to conduct the assessment.

Following the recommendations of the UK HTA program [19], a third of participants in the deliberation and prioritization sessions must be patient representatives. The recruitment of these representatives will begin with a description of the work and the profile of the potential participant, based on criteria developed by the NHS in the UK [20,40]. The collaboration of cancer-related community organizations in this project will make it possible to identify relevant candidates. To maximize the contribution of patient representatives, we will try to recruit individuals who are connected to peer networks and are thus able to suggest a wide corpus of patients. We will also try to respect balance regarding gender and age in each of the groups.

The evaluation of public involvement in HTA prioritization in the UK has demonstrated the importance of providing dedicated staff and regular feedback to support this involvement [19,26,27,40,42]. Therefore, patients will receive training in HTA and evidence-informed healthcare before the prioritization meeting [20]. A member of the research team will also provide ongoing support for patient representatives throughout the process.

### Development of the assessment plan

For the last stage, a working group consisting of various stakeholders, including patients, will be responsible for the refining the topic selected and developing the assessment plan. They will specify the research question, the dimensions to be evaluated, and the strategies to implement in order to conduct this HTA, notably regarding patient involvement in the next stages of the process. The HTA process will then continue under the auspices of the HTA unit of the QUHC, so this study will stop following the development of the assessment plan, but our previous collaborative projects with this HTA unit will have laid the groundwork for pursuing patient involvement at the next stages of the project.

### Evaluation of processes and outcomes associated with patient involvement

The research team will observe and evaluate the process and outcomes associated with patient involvement throughout the project using a participatory evaluation approach [35-38]. Our team has already used this approach successfully in a previous project [33]. A workshop will be conducted at the beginning of the project with knowledge users of the team to reach a consensus on the evaluation objectives and questions. Data collection methods, indicators of effects, outcomes and a data-analysis plan will also be discussed, as well as the roles and responsibilities of each team member. Such an approach is recognized as being the most effective for knowledge translation [43,44]. It helps improve the outcomes of an intervention because the results are consistent with practical information needs of knowledge users and speak to the challenges they have to face [45]. It also allows stakeholders to recognize themselves in the results and recommendations made, which facilitates their use in decision making [45].

To assess interventions, we will consider the criteria related to the processes and those associated with the results or impacts. We will partly apply the model proposed by Rowe [46], which identifies nine criteria for assessing patient involvement: those related to the construction and implementation of the process, i.e. 1) representativeness, 2) independence, 3) early involvement, 4) influence or impact on decision making, 5) transparency; and those related to the process, including 6) accessibility of resources to fulfill its role, 7) task definition, 8) structured decision making and 9) cost-effectiveness. However, a subsequent analysis of these criteria by Rowe and collaborators [47] showed that although they have a certain validity, they are not exhaustive, nor are they necessarily appropriate for all involvement activities or all contexts. The choice of outcome indicators, methods and analysis strategies will be based on discussions with researchers and decision makers, an approach recognized as being the most effective for knowledge translation [43,44].

The evaluation of all interventions will be based mainly on a qualitative approach, through in-depth interviews and observations. The interviews will explore patients’ contributions and their influence on the process and results, as perceived by various stakeholders. Patient satisfaction and perceptions regarding the final list of topics and criteria that justified their choice will be of particular interest to us. The interviews will also allow us to collect suggestions to improve the process. The deliberation sessions will be observed to analyze the course of discussions and decision making. Document analysis will allow comparing the research priorities identified by patient representatives, healthcare professionals, and managers.

The evaluation could contribute to improving current knowledge on the impact of patient involvement in the selection of HTA topics and on the factors influencing this involvement. We will also pay particular attention to the possibility of transposing the interventions implemented to other HTA contexts. The proposed approach is important in order to complete the knowledge translation cycle [48] since the knowledge produced through this project will be useful for the development of future interventions to support patient involvement in HTA.

### Ethical considerations

Ethics approval for the project has been received from the Research Ethics Board of CHU de Québec (approved on September 19, 2013; ethics number C13-08-1761). Interviewees will be asked to complete a consent form presenting the research objectives and information about research implications.

## Discussion

This project will help setting up interventions to promote patient participation in three stages of the HTA process, and assessing the impacts of these interventions on the relevance of the topic prioritized and the assessment plan from the perspectives of patients and other groups involved in the project. This study will complement our previous project, which enabled the involvement of patients in a specific HTA on alternative measures to restraint and seclusion [33]. In that particular project, involvement of patients in the stages of identification, prioritization, and development of the assessment plan was not possible due to the timing of the research. Based on the positive results of this experience, the present project will make it possible to involve patients in the early stages of HTA that have not been considered in our previous work.

Despite various initiatives to involve patients in the HTA stages, assessments of the impact of these initiatives are rather sparse and anecdotal [10]. Evidence on the effectiveness of public participation (including patients) in HTA is only beginning to emerge [14,40]. The project will therefore allow us not only to apply knowledge from other studies in the Canadian context, but also to evaluate proposed interventions, and thus will provide essential knowledge on the effectiveness of the involvement strategies put in place by paying particular attention to the involvement process, including its implementation and follow-up, while considering the views of different stakeholders. If we do not want this involvement to remain anecdotal, it is important for knowledge users to rely on evidence to develop strategies for patient involvement in HTA decision making [49].

### Knowledge translation activities

In this project, knowledge translation is adapted to the local context and to knowledge users’ needs because of the collaborative approach adopted. One of the strategies will consist in presenting progress reports and results periodically to all stakeholders involved in the project. We will also communicate the results of each phase to stakeholders from the regions concerned through the participation of researchers from the team in the HTA Roundtable of the IUHN. Targeted presentations will provide a forum for discussing the research process, data analysis, and interpretation of results. Presentations will also be made to provide information about the process of the project and present its results to a wider audience (Ministry of Health and Social Services, Quebec National Institute of Public Health, Health and Welfare Commission, as well as relevant patient organizations). This will facilitate a consideration of the potential for dissemination and the applicability of the interventions to other organizations interested in HTA. These presentations will be integrated into the regular training activities of the organizations concerned. A brief questionnaire will be distributed to participants after each meeting to assess the relevance of these meetings, the understanding of key messages, and the intention to apply them in their practice.

The research team will work with the HTA Roundtable of the IUHN to share and disseminate lessons learned with the Quebec community of practice in HTA, and other provincial, national and international HTA organizations. To adapt the dissemination of results to different audiences, representatives of all groups involved in the project will participate in the dissemination process. Collaborations with patient groups and community organizations will also facilitate the sharing of results directly with patients.

## Abbreviations

HTA: Health technology assessment; IUHN-LU: Integrated University Health Network (IUHN) of Laval University (LU); QUHC: Quebec University Hospital Centre; CIHR: Canadian Institutes of Health Research.

## Competing interests

The authors declare that they have no competing interests.

## Authors’ contributions

MPG, BC, FL, JG and MD conceived and designed the study and drafted the manuscript. DL, MC and MR participated in designing the study and revised the manuscript. MTP helped to draft the manuscript. All authors read and approved the final manuscript.

## Authors’ information

MPG is Tier 2 Canada Research Chairholder in Technologies and Practices in Health. FL is Tier 2 Canada Research Chairholder in Implementation of Shared Decision Making in Primary Care.

## Pre-publication history

The pre-publication history for this paper can be accessed here:

http://www.biomedcentral.com/1472-6963/14/273/prepub
